# A cross-cohort computational framework to trace tumor tissue-of-origin based on RNA sequencing

**DOI:** 10.1038/s41598-023-42465-8

**Published:** 2023-09-16

**Authors:** Binsheng He, Hongmei Sun, Meihua Bao, Haigang Li, Jianjun He, Geng Tian, Bo Wang

**Affiliations:** 1 School of Pharmacy, Changsha Medical University, Changsha, 410219 People’s Republic of China; 2 Academician Workstation, Changsha Medical University, Changsha, 410219 People’s Republic of China; 3 Department of Medical Oncology, The Cancer Hospital of Jia Mu Si, Jiamusi, People’s Republic of China; 4 Geneis Beijing Co., Ltd., Beijing, 100102 People’s Republic of China; 5 Qingdao Genesis Institute of Big Data Mining and Precision Medicine, Qingdao, 266000 Shandong People’s Republic of China

**Keywords:** Cancer, Computational biology and bioinformatics

## Abstract

Carcinoma of unknown primary (CUP) is a type of metastatic cancer with tissue-of-origin (TOO) unidentifiable by traditional methods. CUP patients typically have poor prognosis but therapy targeting the original cancer tissue can significantly improve patients’ prognosis. Thus, it’s critical to develop accurate computational methods to infer cancer TOO. While qPCR or microarray-based methods are effective in inferring TOO for most cancer types, the overall prediction accuracy is yet to be improved. In this study, we propose a cross-cohort computational framework to trace TOO of 32 cancer types based on RNA sequencing (RNA-seq). Specifically, we employed logistic regression models to select 80 genes for each cancer type to create a combined 1356-gene set, based on transcriptomic data from 9911 tissue samples covering the 32 cancer types with known TOO from the Cancer Genome Atlas (TCGA). The selected genes are enriched in both tissue-specific and tissue-general functions. The cross-validation accuracy of our framework reaches 97.50% across all cancer types. Furthermore, we tested the performance of our model on the TCGA metastatic dataset and International Cancer Genome Consortium (ICGC) dataset, achieving an accuracy of 91.09% and 82.67%, respectively, despite the differences in experiment procedures and pipelines. In conclusion, we developed an accurate yet robust computational framework for identifying TOO, which holds promise for clinical applications. Our code is available at http://github.com/wangbo00129/classifybysklearn.

## Introduction

Carcinoma of unknown primary (CUP) is a type of metastatic cancers with unknown cancer origin. CUP accounts for 3–5% of all cancer incidences in the United States^1^. Although there is no drug specifically approved for CUP, multiple guidelines recommend treating this disease using multi-agent cytotoxic chemotherapy^2,3^. However, the responses of CUP patients to non-targeted chemotherapies are poor with a 5-year survival rate around 11%^4^.

In order to solve this problem of identifying the tissue-of-origin (TOO), several diagnostic methods have been proposed in the past decades. From 1980 to 2010s, immunohistochemistry is the mainstream method to identify cancer primary tissue^5–12^. However, this method is labor-intensive, requires highly skilled physicians, and has varying accuracy rates in predicting TOO for different cancer types^13^. Although imaging techniques such as PET/CT and ultrasound have been utilized for assisting in clinical diagnosis of CUP^14–18^, their diagnostic accuracies vary from ~ 30 to ~ 90%, which is not high enough for safe clinical usage. Thus, novel diagnostic methods are needed to address this issue.

Recently, with the advancement of sequencing techniques, omics data including RNA expression profile^19–27^, mutation profile^28,29^, copy number profile and methylation profile were used in the diagnosis of CUP. The assumption for inferring TOO using the omics data is that the metastatic site retains the molecular characteristics of the primary site^30^. For example, Liu et al. achieved an accuracy of 81% using mutation profile across 13 cancer types^28^. Ma et al. achieved an accuracy of 84% across 39 cancer types using expression profile^24^. There are also methods combining multiple types of omics data. For example, He et al. inferred TOO by integrating the features from RNA expression and DNA somatic mutation^31^. Liu et al. evaluated the potential for identifying TOO using methylation, expression and mutation data, finding that methylation could achieve similar accuracy as expression data for inferring TOO^32^. However, since the methylation data is more expensive than other omics data such as expression profiles, inferring CUP using expression profile is currently the recommended approach.

For obtaining gene expression profile, RT-PCR, micro-array and RNA-seq were majorly used. For TOO tracing using RT-PCR, Ma et al. collected 578 labeled samples covering 39 tumor types, including 75% primary tumors and 25% metastatic tumors. The dataset was split to 466-sample dataset (frozen) and 112-sample test set (FFPE) according to the sample type. A 92-gene list was used for inferring TOO, and k-nearest neighbor algorithm (KNN) (k = 5) was applied to the problem, reaching an accuracy of 84% in the leave-one-out cross validation. The result also showed there was no difference in the accuracies on predictions of primary or metastatic tumor^24^. Using micro-array, Bloom et al. combined the cDNA and oligonucleotide platform with artificial neural network (ANN) to trace the primary tumor origin^26^, obtaining an accuracy of 83–88% on different platforms. Xu et al. reported a multiple-platform 154-gene panel based on TCGA RNA-seq data to detect the primary origin of metastatic tumors. They selected the 154 genes by recursive feature selection and trained a classifier based on support vector machine (SVM), achieving an overall accuracy of 92%^33^. For TOO tracing using RNA-seq, Liang et al. developed a TOO classifier on TCGA data based on Naïve Bayes algorithm, achieving an accuracy of 91%^34^. Li et al. used TCGA RNA-seq data as the training set and achieved an accuracy of 96.1% for cross-validation, and an accuracy of 83.5% for an independent GEO dataset^35^. Deep learning-based methods were also used to infer TOO, such as Grewal et al.’s neural network achieving a 99% accuracy in a 126-sample dataset and an 86% accuracy in a 201-sample dataset^36^. He et al. developed a neural network for predicting TOO using 150 genes at a 94.87% accuracy^27^. Zhao et al. developed pipeline by log transformation followed by an 1D-inception structure for inferring TOO, achieving an accuracy of 98.54% in the cross-validation phase, surpassing most methods before^37^.

Although TOO inference methods usually perform well in cross-validation, they are often insufficient when tested on independent samples, particularly those with cancer metastasis. Furthermore, a comprehensive comparison on the effects of different gene normalization methods, feature selection techniques and classification algorithms is yet to be conducted. Here, we designed a computational framework to infer TOO based on machine learning integrating normalization, feature selection, training and testing processes. We also conducted a comprehensive analysis of different normalization, feature selection and classification methods. Based on the analysis, we proposed a model that employs the most effective combination of normalization, feature selection and classification method. Finally, we evaluated the performance of our trained model on independent datasets.

## Results

### Dataset preparation

We collected RNA-seq data from two sources in this study. First, we collected a 10,304-sample data from The Cancer Genome Atlas (TCGA) and further split it into a 9911-sample primary dataset and 393-sample metastatic dataset, as described in Materials and Methods. For independent validation, We obtained a 1988-sample dataset from The International Cancer Genome Consortium (ICGC)^38^. We present the details for all datasets used in Table 1.

**Table 1:** Datasets used in this study.

Abbreviation	TCGA primary	TCGA metastatic	ICGC	Cancer name
ACC	79	0	0	Adrenocortical carcinoma
BLCA	414	0	0	Bladder urothelial carcinoma
BRCA	1102	7	50	Breast invasive carcinoma
CESC	304	2	0	Cervical squamous cell carcinoma and endocervical adenocarcinoma
CHOL	36	0	0	Cholangiocarcinoma
COAD	478	1	0	Colon adenocarcinoma
DLBC	48	0	107	Lymphoid neoplasm diffuse large B-cell lymphoma
ESCA	161	1	0	Esophageal carcinoma
GBM	156	0	0	Glioblastoma multiforme
HNSC	500	2	40	Head and neck squamous cell carcinoma
KICH	65	0	0	Kidney chromophobe
KIRC	538	0	136	Kidney renal clear cell carcinoma
KIRP	288	0	0	Kidney renal papillary cell carcinoma
LAML	151	0	323	Acute myeloid leukemia
LGG	511	0	0	Brain lower grade glioma
LIHC	371	0	606	Liver hepatocellular carcinoma
LUAD	533	0	0	Lung adenocarcinoma
LUSC	502	0	0	Lung squamous cell carcinoma
MESO	86	0	0	Mesothelioma
OV	374	0	111	Ovarian serous cystadenocarcinoma
PAAD	177	1	389	Pancreatic adenocarcinoma
PCPG	178	2	0	Pheochromocytoma and paraganglioma
PRAD	498	1	169	Prostate adenocarcinoma
READ	166	0	0	Rectum adenocarcinoma
SARC	259	1	57	Sarcoma
SKCM	103	367	0	Skin cutaneous melanoma
STAD	375	0	0	Stomach adenocarcinoma
TGCT	150	0	0	Testicular germ cell tumors
THCA	502	8	0	Thyroid carcinoma
THYM	119	0	0	Thymoma
UCEC	551	0	0	Uterine corpus endometrial carcinoma
UCS	56	0	0	Uterine carcinosarcoma
UVM	80	0	0	Uveal melanoma
Sum	9911	393	1988	Sum of all cancers

The TCGA primary dataset covering 33 main cancers (all cancer abbreviations are supplied in Table 1) were collected. We also merged the two cancers, colon adenocarcinoma (COAD) and rectum adenocarcinoma (READ) to COADREAD, since they have similar molecular profiles^39^. As a result, we used 32 cancer types from TCGA. The TCGA dataset contains 9911 primary tumor samples covering all 32 cancers and 393-metastatic tumor samples covering 11 cancers. The ICGC dataset contains 1988 samples and covered 10 cancers. We used the TCGA primary dataset to train our model, and used the TCGA metastatic dataset and the ICGC dataset to access our model.

### Combinations of preprocessing, feature selection and classification were assessed

In this study, we systematically researched the algorithms employed in each necessary step for detecting TOO using the TCGA primary dataset were used for investigation. We evaluated two preprocessing methods, l1-normalization (like TPM) and standardization after log2 transformation^37^. We also investigated two feature selection methods, random forest and logistic regression, and included random selection for baseline comparison. We consider feature numbers selected by each method as an important factor for feature selection. Finally, we applied three classification method including logistic regression, random forest and KNN. All the methods used in this study were listed in Table 2.

**Table 2:** Methods used for different steps.

Step	Methods	Abbreviation in Figure
Preprocessing	L1-normalization	L1-normalization
	Standardization after log2 transformation	Standardization after log2 transformation
Feature selection	Random forest	rf
	Logistic regression	Logistic
	Random selection	Random
Gene number	50 (5 for logistic regression)	Not applicable
	100 (10 for logistic regression)	
	200 (20 for logistic regression)	
	400 (40 for logistic regression)	
	800 (80 for logistic regression)	
Classification	Random forest	rf
	Logistic regression	lr
	K-nearst neighbor	knn
	Support vector machine	svm

**Figure 1:**
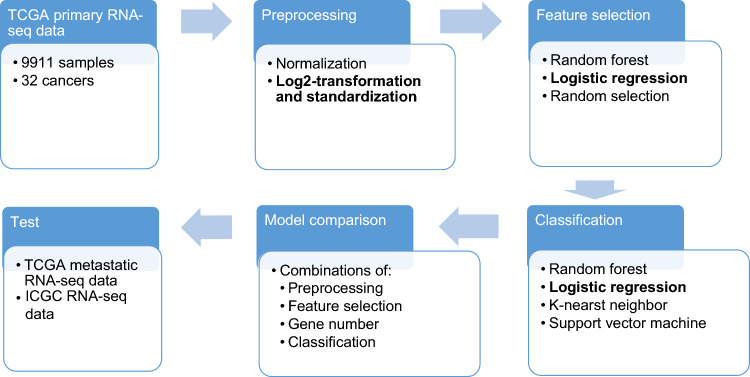
Datasets and flowchart of this work. TCGA primary dataset was used to evaluate the different combinations of preprocessing, feature selection and classification methods. The best combination will be used to train on the TCGA primary dataset and the trained model will be used to test on independent datasets.

The training data was used to test the combinations of preprocessing, feature selection and classification methods using tenfold cross validation. The best combination was used to train a model on the TCGA primary dataset. The trained model was tested on the independent test datasets. A schematic diagram of our approach work is shown in Fig. 1.

### Logistic regression performed best in training dataset during cross validation

**Figure 2:**
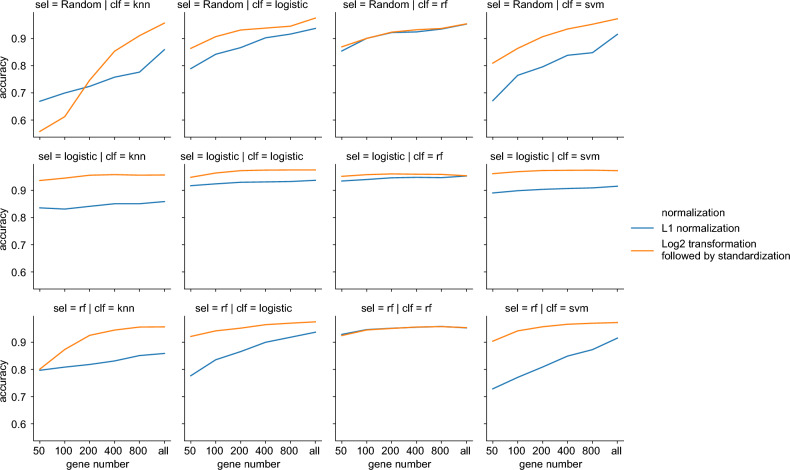
Accuracies for different combinations of preprocessing, feature selection and classification methods using tenfold cross validation on the training dataset. *sel* feature selection method, *clf* classification method; the other abbreviations were mentioned in Table 2. Python package seaborn version 0.9.0 was used to plot this figure.

We evaluated the tenfold cross validation accuracy on the training dataset to assess the effectiveness of each step. We created a plot of the accuracies of all possible combinations in Fig. 2. The optimal combination included standardization after log2 transformation, feature selection by logistic regression (using 80 genes for each type of cancer) and classification by logistic regression, which achieved an accuracy of 97.50%. The precisions for each cancer ranged from 79.41 (CHOL) to 100.00% (MESO, LAML, UVM, THYM, TGCT, LGG, PRAD, GBM, OV, THCA and SKCM). The recalls for each cancer ranged from 75.00 (CHOL) to 100.00% (THCA, GBM, UVM, PRAD, THYM and LAML). The specificities for each cancer ranged from 99.67 (STAD) to 100.00% (UVM, THYM, THCA, GBM, SKCM, OV, MESO, LGG, LAML, TGCT and PRAD). Besides, we supplied accuracies, precisions, recalls and specificities for all combinations in Supplementary Table 1 sorted by accuracy.

The gene number is a significant factor affecting the classification performance, while the random forest algorithm was relatively insensitive to the gene number, demonstrating the robustness of ensemble learning. Log2 transformation, followed by standardization, was superior to l1 normalization in most cases. This may be caused by that the optimizer perform better when data is normally distributed. Random selection for feature selection could also work well, except for the KNN method.

It is worth noting that logistic regression was the feature selection method in six of the top 10 combinations (Supplementary Table 1). Random forest was the feature selection method in the 9th and 10th combinations and used more genes than the 6th and 7th combinations, despite using the same classification methods. This suggests that there are associations between log2-transformed expression profiles and cancer types. The top 10 combinations contained only logistic regression and SVM classification methods, indicating strong associations between log2-transformed expression profiles and cancer types. Interestingly, even when using all genes without feature selection, the logistic regression could only reach exactly the same accuracy as using feature selection, indicating the redundancy in features.

The standardization after log2 transformation was the best preprocessing method in most cases, as shown in Fig. 2. The reasons might be due to the facts that (a) the expression values were scaled to the same scale after log2 transformation, eliminating extreme values and (b) the normal distribution might help the optimizers. KNN performed as well as other methods after log2 transformation and standardization. Furthermore, random forest is able to perform well even without log2 transformation and standardization, showing the tree method is robust to the distribution of the input data.

**Figure 3:**
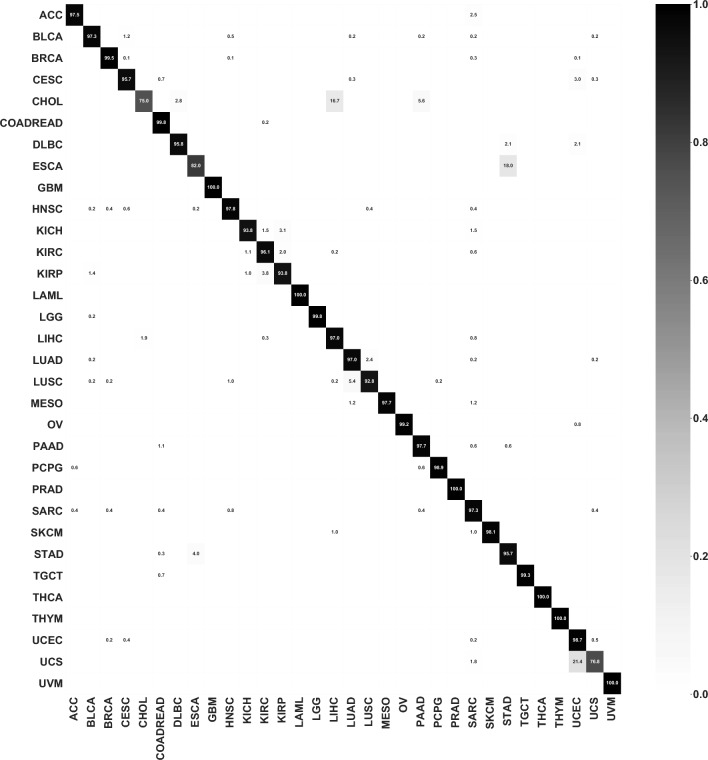
Confusion matrix for the best combination, which consists log2 transformation followed by standardization, feature selection and classification by logistic regression. The numbers shown in the figure are the classification prediction percentages for each cancer. For each row, the percentages sum to 1.

We plotted the confusion matrix for the best combination in Fig. 3. The diagonal showed the percentage of the correctly classified ratio for each cancer type. The majority of the samples were classified correctly. CHOL, ESCA and UCS tended to be misclassified as their adjacent cancers, LIHC, STAD and UCEC, which may be due to their close development and spatial relationship.

### Combination of logistic regression and SVM allows gene set to be narrowed down

When the standardization after log2 was used, we noticed a high 96.14% accuracy was achieved using only 5 genes per cancer when we use logistic regression to select genes for each cancer and SVM as the classification algorithm. This is comparable to other combinations using more genes. Random forest using 20 genes selected from logistic regression achieved 96.04% accuracy. Even for logistic regression itself using 5 genes per cancer could only achieve 94.80% accuracy. To investigate whether we could use less genes for the combination, we narrowed down the gene set for logistic regression to 1 to 4 for each cancer type. Accuracies of 88.16%, 93.48%, 95.08% and 95.69% were achieved separately for 1 to 4 genes per cancer. We noticed that even using 1 gene per cancer, SVM’s classification accuracy (88.16%) is comparable to that of selecting 100 genes in total by random forest and classifying by KNN (87.28%). In summary, the selection of methods and classification algorithms can significantly impact the accuracy of predictions.

### Informative genes were selected by logistic regression

We conducted feature selection for all training samples by performing log2 transformation followed by standardization using logistic regression. 80 genes were selected for each cancer (see Supplementary Table 2 for details).

**Figure 4:**
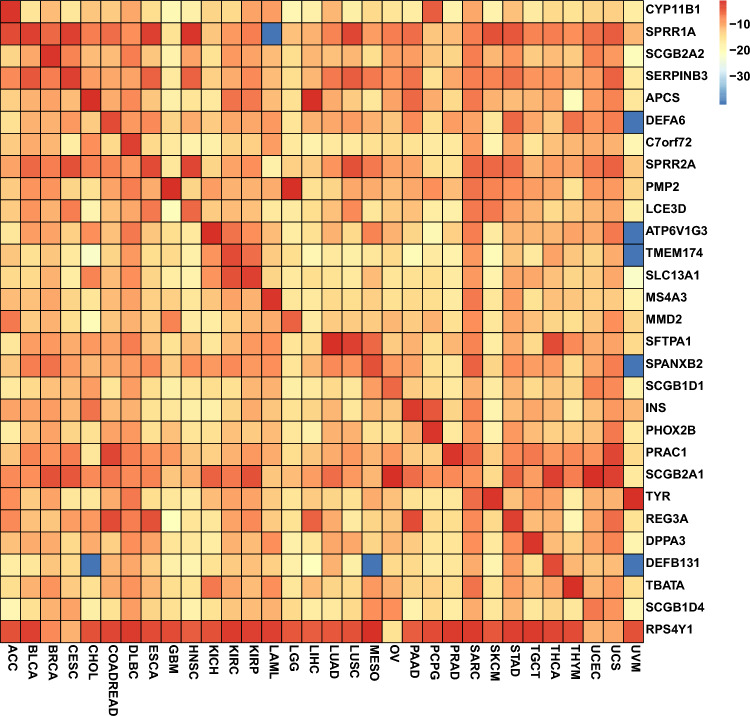
The heatmap for average expression value (log2 transformed) for top-1 selected gene per cancer for the training dataset. Most values on the diagonal demonstrated high-level expression and most values off the diagonal were of low expression level, showing that the feature selection process tends to select the most informative genes for each cancer. R package pheatmap was used to plot the heatmap.

The top gene, characteristic of each cancer, was identified and combined into a set for expression level visualization. The log2 transformed average expression value for selected genes in different cancers were represented on a heatmap, shown in Fig. 4. The on-diagonal expression values were higher than the off-diagonal values. For example, CYP11B1 is highly expressed in adrenocortical carcinoma (ACC), which has been reported to be able to differentiate ACC from Cushing Syndrome^40^. The results indicated the logistic regression has the potential to detect the highly informative genes while comparing each one-vs-all classification. Additionally, some marker genes, such as SPRR1A in acute myeloid leukemia (LAML) and DEFA in uveal melanoma (UVM), have low expression levels in some cancers, revealing how marker genes can provide more relevant information.

Gene sets for each cancer were analyzed to look for similarities, and we found that several gene sets overlapped. For example, 53 genes were common to both the cholangiocarcinoma (CHOL) and liver hepatocellular carcinoma (LIHC) gene sets, which explained the misclassification between these two cancers.

**Figure 5:**
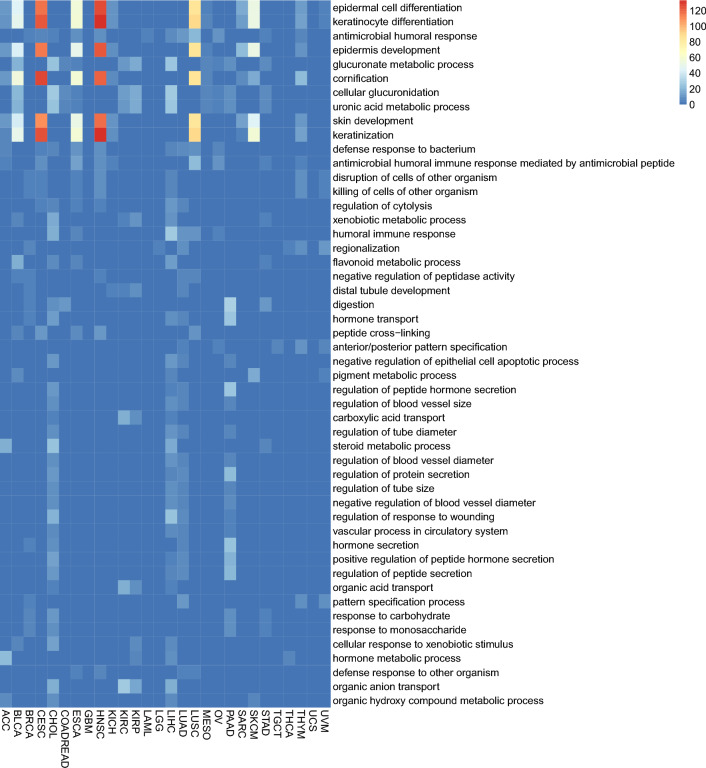
The gene ontology enrichment for the selected 80 genes for each cancer. The color indicates the − log (adjusted *p* value) for each enrichment. Dark blue indicates non-enrichment. Only the top-50 frequent terms in all cancers were shown.

We further examined gene functions of all 80-gene sets using enrichment analysis. The results showed a high degree of enrichment in common human organ developmental processes, such as keratinocyte differentiation, epidermal cell differentiation, and epidermis development, as shown in Fig. 5. Additionally, some gene sets were enriched in specific organ development, including digestion, and skin development and distal tubule (Supplementary Figs. 1, 2). Interestingly, our analysis revealed that genes selected for CHOL and LIHC were enriched in similar pathways, suggesting that these two types of cancer could share similar developmental processes, leading to a similar expression level.

**Figure 6:**
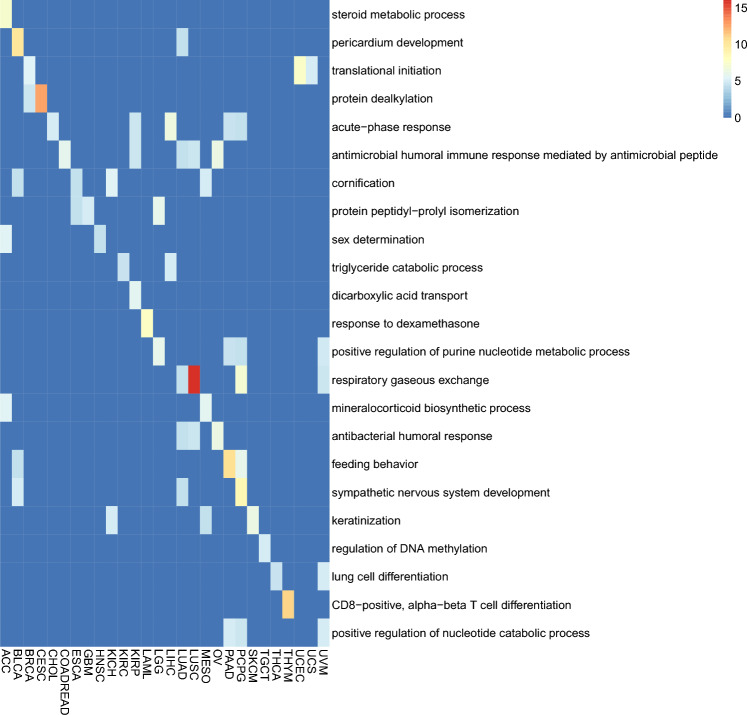
The gene ontology enrichment for the selected 5 genes for each cancer. The color indicates the − log (adjusted p value) for each enrichment. Only the most significantly enriched pathway for each cancer were shown.

We also performed the enrichment analysis on the top-5 genes from each cancer to find the functions of the core genes that take the major effect in the predictions. We demonstrated the most significant pathways from each cancer in Fig. 6. The most significant pathways were tissue-specific. For example, steroid metabolic process was enriched in ACC, corresponding to adrenal cortex secreting adrenocortical hormones. We also noticed a significant enrichment of respiratory gaseous exchange in both lung adenocarcinoma (LUAD) and lung squamous cell carcinoma (LUSC), indicating both cancers were related to breath.

### The model trained from TCGA dataset performed well in independent datasets

**Figure 7:**
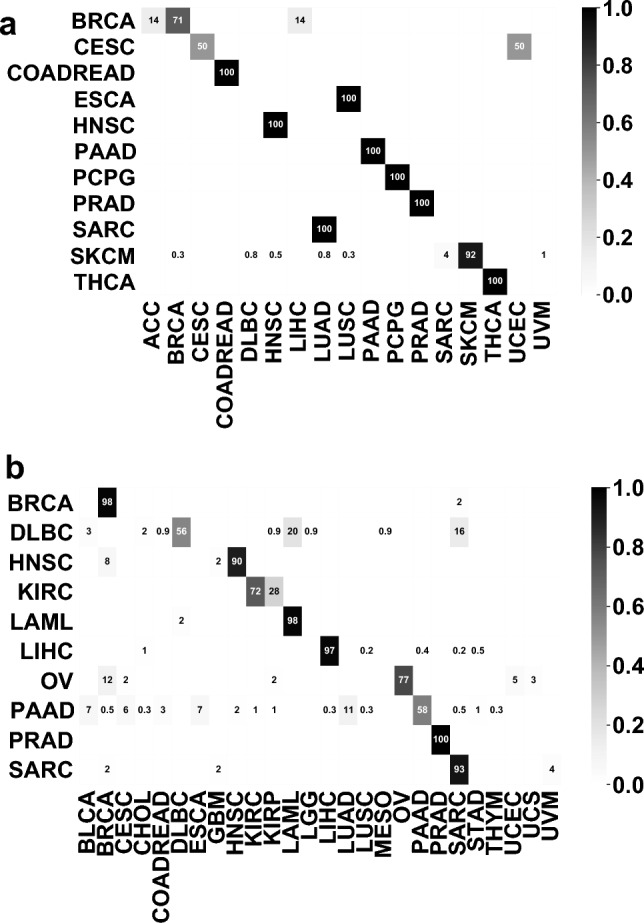
Confusion matrices on (**a**) TCGA metastatic dataset and (**b**) ICGC dataset. For the ICGC dataset, the model was a re-trained model using 9180 overlapping genes between TCGA dataset and ICGC dataset.

To verify our framework in independent datasets, all 80-gene sets for 32 cancer types were combined to create a comprehensive 1356 gene set for further training and used logistic regression as the classification algorithm. We tested our model on 2 independent datasets: (1) the metastatic dataset from TCGA; (2) the non-TCGA ICGC dataset. The model trained from TCGA primary tumor dataset using 1356 genes achieves a 91.09% accuracy on the metastatic dataset from TCGA. We plotted the confusion matrix for the dataset in Fig. 7a and included the prediction probabilities for all samples in Supplementary Table 3. Most incorrect classifications were SKCM samples. We hypothesize the discrepant distribution (103 in the training set and 367 in the test set) between the two datasets may have resulted in an inadequate training of our model. One case of cervical squamous cell carcinoma and endocervical adenocarcinoma (CESC) was erroneously classified as Uterine Corpus Endometrial Carcinoma (UCEC), potentially due to their similar tissue of origin.

To account for the lack of full consistency in gene sets between the TCGA and ICGC datasets, we initially created an overlapping gene set of 9180 genomic features. Using this 9180-gene set, we conducted preprocessing, feature selection, and model training on the TCGA primary datasets resulting in the identification of 80 genes by feature selection. Feature selection was applied, allowing for the selection of 474 genes for the final model training. This set of 474 genes was integrated into a logistic regression model, which was used to test the ICGC dataset with 82.67% accuracy. The confusion matrix for the dataset is displayed in Fig. 7b, and the prediction probabilities for all samples is supplied in Supplementary Table 4. The model produced some erroneous classifications, including the misclassification of lymphoid neoplasm diffuse large B-cell lymphoma (DLBC) samples as acute myeloid leukemia (LAML), and misclassification of pancreatic adenocarcinoma (PAAD) as other types of cancers. These misclassifications could be attributed to the similarities between the cell types of misclassified cancers. For example, DLBC and LAML both originate from blood forming cells, and PAAD, LUAD and BLCA originates from the glandular cells. The other misclassification of PAAD samples may have resulted from racial differences and technical differences from the ICGC TCGA, such as experimental and expression-calling pipeline differences, since ICGC collected datasets from multiple countries.

## Discussion

In this study, we designed a computational framework including the data preprocessing, feature selection and classification in one tool for TOO inference. Besides, we thoroughly investigated the impact of preprocessing methods, feature selection methods and classification methods on the predication accuracy of tissue-of-origin inference. Our study showed that log2 transformation and standardization provide an optimal starting point for preprocessing for RNA-seq data. Traditional machine learning methods, such as logistic regression yielded similar accuracies to deep learning approaches when using only 1000 genes^27,37^. The robustness of our framework was further indicated by the performance on two independent datasets, which achieved accuracy rates of 91.09% for the TCGA metastatic dataset and 82.67% for the ICGC dataset. These observations demonstrate the efficacy and importance of our computational framework for TOO inference.

There are some limitations to our study. First, we did not explore all possible combinations of the steps. For instance, we did not use quantile normalization, which is a popular method in expression profile, because the method was conducted within one dataset instead of one sample. Additionally, feature selection methods, such as correlation-based methods^27^ or the minimum Redundancy-Maximum Relevance (mRMR) algorithm were not compared. Moreover, gradient boosting decision tree (GBDT)-based methods^35^ and deep learning algorithms^27,37,41,42^ might further enhance the prediction accuracy. Though we achieved a similar accuracy rate of 98.54% for cross-validation as Zhao’s study^37^, we failed to achieve the same level of accuracy (96.70%) for the same TCGA metastatic dataset for independent test. Therefore, we suggest utilizing neural networks like 1-D convolutional networks to improve our predictive result. Furthermore, we suggest exploring classification methods with complex structures, such as multi-layer neural networks, and integrating additional data types, such as histopathological image, which are regularly used in cancer diagnosis and prognosis prediction^43–46^. Although recently developed TOO-inferring medical image-tools show promise^47^, more work in this area is necessary to utilize multi-omics for a higher accuracy in TOO inferring.

Secondly, it is unclear if our framework could infer the subtype of cancer origin. As stated by Zhao et al., small sample number was a barrier for neural networks to learn more information^37^. Conventional machine learning algorithms have less parameters than neural networks. Hence, our framework might be suitable for inferring TOO subtype.

Thirdly, our work did not differentiate FFPE and frozen samples as distinct datasets, as pointed out by Ma et al.^24^. Moreover, we did not compare the performance of our model between different tumor grade levels. Further tuning of our model may be necessary if sample preservation method or tumor grade level were taken into account.

Finally, to make our work medically applicable, in-house RNA-seq data is necessary. Further efforts are required to adjust the parameters per our data. As mentioned above, logistic regression can predict TOO using an expression profile covering 1356 genes. We look forward to utilize sequencing techniques such as capture that sequence specific genes to reduce the costs for the experiment^48–51^.

## Conclusion

This study implemented a machine learning framework to identify the primary origin of tumor tissue using RNA sequencing expression profiles. Comparing different methods for preprocessing, feature selection and classification, we determined that log2 transformation and standardization was superior than normalization methods that express values as a proportion. We found that logistic regression performs well in feature selection and classification for this task. Furthermore, we found that predicting with using 1356 genes as features resulted in a relatively high accuracy for predicting the origin of the primary cancer site. This work suggests the RNA-seq and machine learning algorithms might be used in clinical practice when other pathological methods fails to determine the primary origin site of certain cancers.

## Materials and methods

### Data preparation

The TCGA RNA-seq data were downloaded from TCGA Data Portal (https://portal.gdc.cancer.gov/). The ICGC RNA-seq data were downloaded from Data Portal (https://dcc.icgc.org/releases/release_28/Projects/) by searching the keyword “exp_seq”. To avoid information leakage, the ICGC samples that also showed up in TCGA were not included for ICGC dataset. For the TCGA data, we removed all the metastatic tumors in the TCGA dataset for test set by checking TCGA identifier, leaving the samples with primary tumors (i.e., 01 and 03 for the 4th field) as the training dataset and metastatic tumors (i.e., 06 for the 4th field) as the test dataset. For all datasets, the TPM value of each sample and each gene from were extracted, generating a M × N matrix where M is the number of the sample number and N is the number of the gene number. All the samples were labeled by its cancer type.

### Normalization by l1 normalization

For one sample, the l1 normalization will sum all expression values for all expression values as the denominator. The expression values will all be scaled by this denominator, i.e.

$$G=g/\left(\sum_{i=1}^{n}g\right),$$

where $G$ is the expression value after normalization, $g$ is the expression value before normalization and $n$ is the total number of genes of this sample.

### Normalization by log2 transformation followed by standardization

For one sample, all expression values will log2-tranformed. To avoid log2(0) error, 1e−6 was added to all expression values before log2. The expression values will all be scaled by this denominator, i.e.

$$z = (x - u) / s,$$

where $u$ is the mean of the expression values and $s$ is the standard deviation of the expression values.

### Feature selection by random forest

For selecting features using random forest^52^, a random forest model was trained on all genes. The base estimator number was set to 2000 for the random forest classifier. For each decision tree, sub-samples are drawn with replacement by bootstrapping method. Each decision tree will use up to $\sqrt{selected\, gene\, number}$ genes. Gini impurity was used to find the best split point and feature. The feature importance was used to sort the genes and the top N genes were selected as final features.

### Feature selection by logistic regression

First, a multinomial logistic regression model was trained using all data. The l2 penalty was used for regularization and regularization strength was set to 1e−4 (i.e., C = 10,000 for scikit-learn). Then, for each cancer, the weights for all genes for sorted by absolute value. To select N genes for each cancer, the top-ranked N genes were first selected as the genes for classifying this cancer. The selected N genes for all cancers were combined as the final features. For logistic regression, we divided 10 for the feature selection for each cancer.

### Functional annotation

For the analysis of biological significance, the functions were annotated for the specific gene set. Gene ontology^53,54^ was used as the database for the enrichment analysis. Genes were clustered by R package clusterProfiler^55^. The visualization was done by R package ggplot2^56^.

### Cross validation

In a *N*-fold cross validation (where *N* is an integer), all the samples were stratified into *N* subsets by different random seeds. And the algorithm was repeated *N* times. During each repeat, one of the *N* subsets was used as the test set and the other *N* − 1 subsets were consolidated to a training set. Features that were selected within the training set were used to train a model. The test set was then used to evaluate the model. Then the average error across all *N* trials was computed.

### Classification by random forest and logistic regression

We used the default parameters in random forest and logistic regression.

### Classification by support vector machine

For the multi-class classification based on SVM, the one-vs-all strategy and rbf kernel were used. For regularization, l2 penalty was used and the inverse regularization parameter C was set to 10,000 for scikit-learn implementation.

All above mentioned feature selection and classification methods were implemented using scikit-learn package^57^.

### Accuracy visualization for all combinations

To plot the accuracies for all combinations, functions of FacetGrid from package seaborn version 0.9.0 was used^58^.

### Heatmap visualization

To plot the heatmaps, the R package pheatmap version 1.0.12 was used^59,60^. Before plotting, the expression values were first added 1e−12 and transformed by log2.

### Supplementary Information

Supplementary Figure 1.

Supplementary Figure 2.

Supplementary Table 1.

Supplementary Table 2.

Supplementary Table 3.

Supplementary Table 4.

## Data Availability

The data that support the findings of this study are available from public databases, TCGA (https://portal.gdc.cancer.gov/) and ICGC (https://dcc.icgc.org/releases/release_28/Projects/).
