# Supplementary material for: A cross-cohort computational framework to trace tumor tissue-of-origin based on RNA sequencing
Source: Sci Rep. 2023 Sep 16;13:15356. doi: 10.1038/s41598-023-42465-8 (PMC10505149; doi:10.1038/s41598-023-42465-8)
Supplement: Supplementary file 1 — Supplementary Figure 1. [file 41598_2023_42465_MOESM1_ESM.pdf]

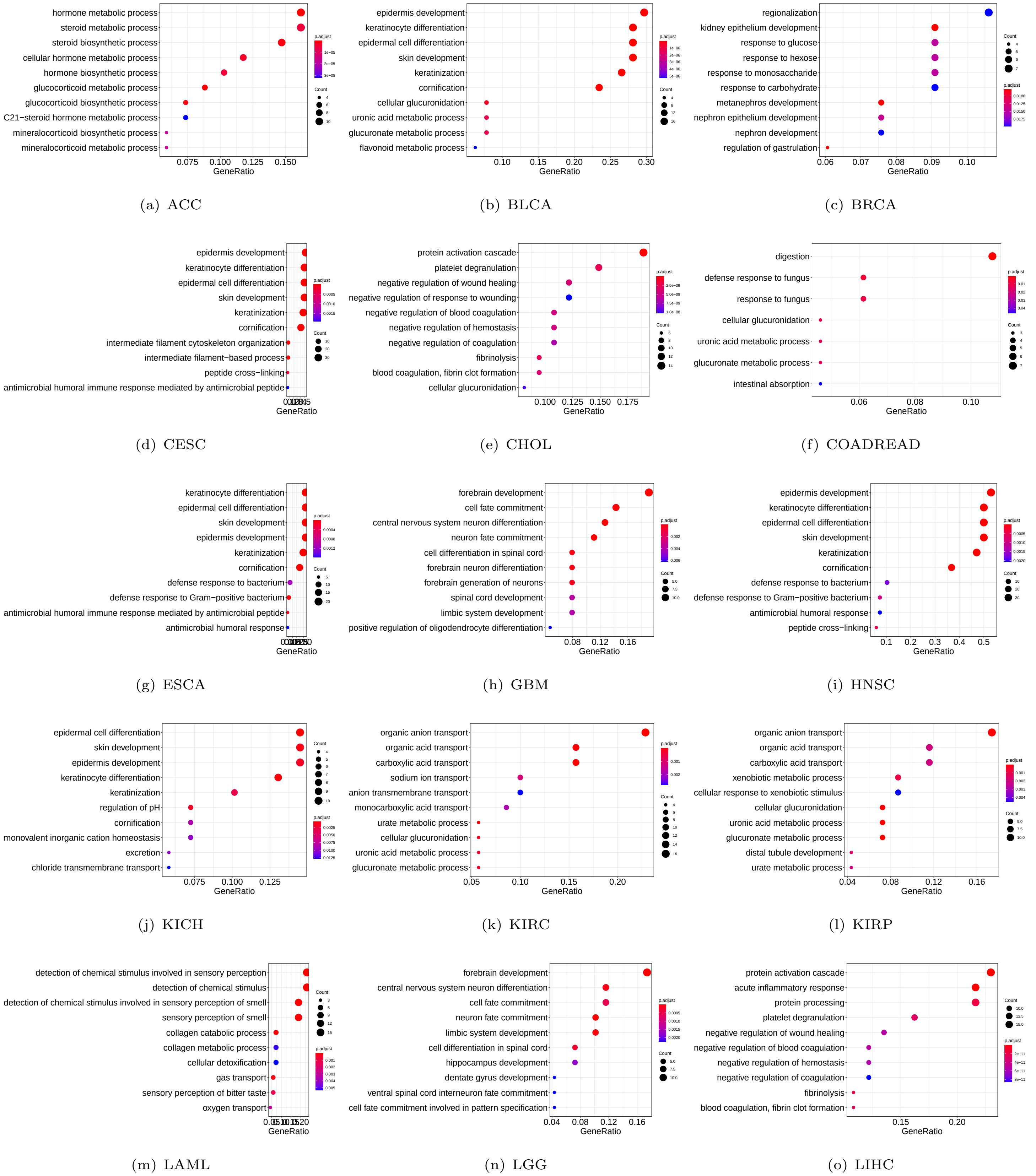

Supplementary Figure 1. Biological process enrichments for select gene sets for all cancer types (part 1).
