# Supplementary material for: A cross-cohort computational framework to trace tumor tissue-of-origin based on RNA sequencing
Source: Sci Rep. 2023 Sep 16;13:15356. doi: 10.1038/s41598-023-42465-8 (PMC10505149; doi:10.1038/s41598-023-42465-8)
Supplement: Supplementary file 2 — Supplementary Figure 2. [file 41598_2023_42465_MOESM2_ESM.pdf]

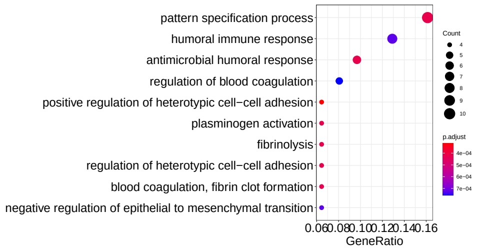

(a) LUAD

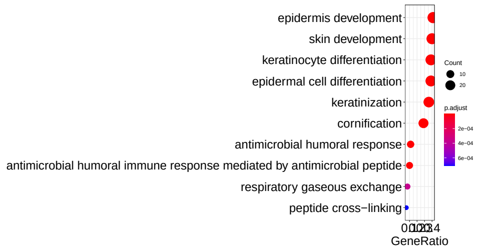

(b) LUSC

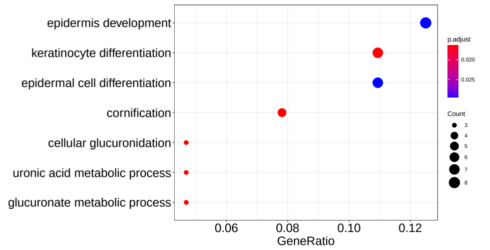

(c) MESO

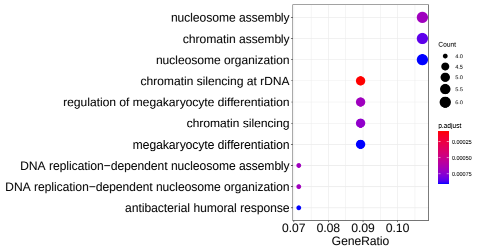

(d) OV

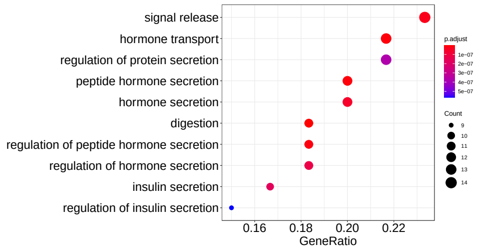

(e) PAAD

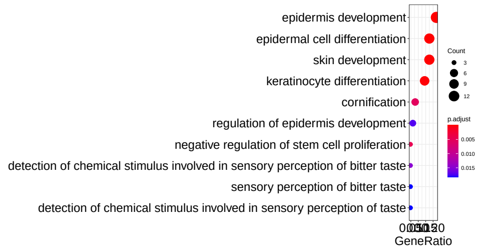

(f) SARC

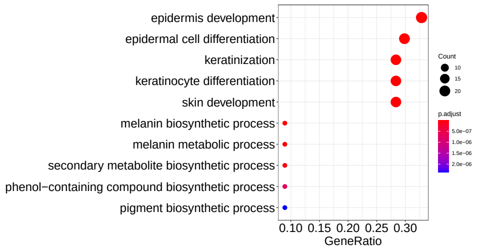

(g) SKCM

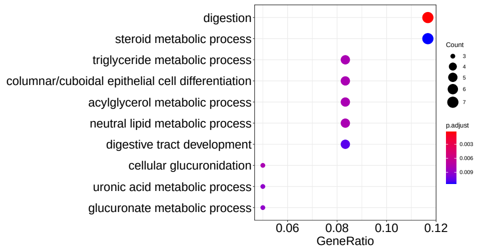

(h) STAD

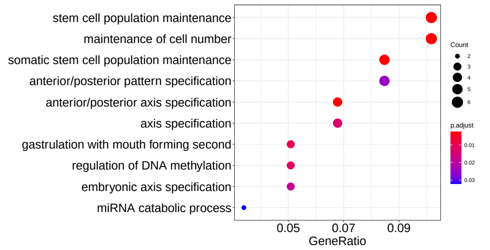

(i) TGCT

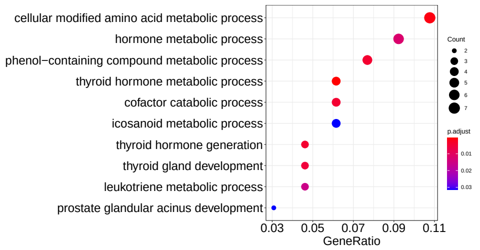

(j) THCA

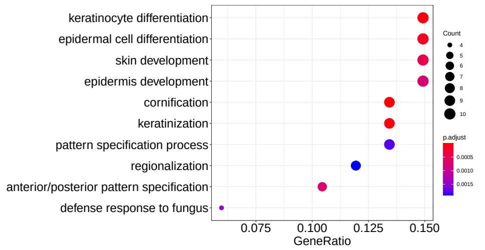

(k) THYM

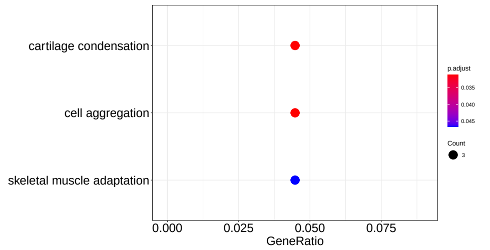

(l) UCS

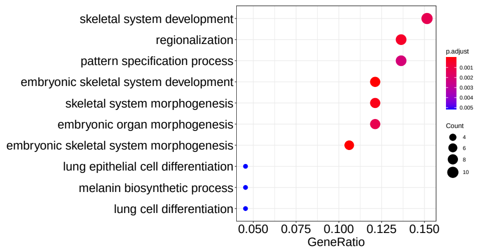

(m) UVM

Supplementary Figure 2. Biological process enrichments for select gene sets for all cancer types (part 2).
